# Reductions in the dietary niche of southern sea otters (*Enhydra lutris nereis*) from the Holocene to the Anthropocene

**DOI:** 10.1002/ece3.6114

**Published:** 2020-03-10

**Authors:** Emma A. Elliott Smith, Martin Tim Tinker, Emily L. Whistler, Douglas J. Kennett, René L. Vellanoweth, Diane Gifford‐Gonzalez, Mark G. Hylkema, Seth D. Newsome

**Affiliations:** ^1^ Department of Biology University of New Mexico Albuquerque NM USA; ^2^ Nhydra Ecological Consulting St Margaret’s Bay Nova Scotia Canada; ^3^ Department of Ecology and Evolutionary Biology University of California Santa Cruz Santa Cruz CA USA; ^4^ Department of Anthropology Washington State University Pullman WA USA; ^5^ Department of Anthropology University of California Santa Barbara CA USA; ^6^ Department of Anthropology California State University Los Angeles Los Angeles CA USA; ^7^ Department of Anthropology University of California Santa Cruz Santa Cruz CA USA; ^8^ California Department of Parks and Recreation Santa Cruz CA USA

**Keywords:** amino acid stable isotope analysis, historical ecology, marine ecology, marine mammal conservation, stable isotopes, zooarchaeology

## Abstract

The sea otter (*Enhydra lutris*) is a marine mammal hunted to near extinction during the 1800s. Despite their well‐known modern importance as a keystone species, we know little about historical sea otter ecology. Here, we characterize the ecological niche of ancient southern sea otters (*E. lutris nereis*) using δ^13^C analysis and δ^15^N analysis of bones recovered from archaeological sites spanning ~7,000 to 350 years before present (*N* = 112 individuals) at five regions along the coast of California. These data are compared with previously published data on modern animals (*N* = 165) and potential modern prey items. In addition, we analyze the δ^15^N of individual amino acids for 23 individuals to test for differences in sea otter trophic ecology through time. After correcting for tissue‐specific and temporal isotopic effects, we employ nonparametric statistics and Bayesian niche models to quantify differences among ancient and modern animals. We find ancient otters occupied a larger isotopic niche than nearly all modern localities; likely reflecting broader habitat and prey use in prefur trade populations. In addition, ancient sea otters at the most southerly sites occupied an isotopic niche that was more than twice as large as ancient otters from northerly regions. This likely reflects greater invertebrate prey diversity in southern California relative to northern California. Thus, we suggest the potential dietary niche of sea otters in southern California could be larger than in central and northern California. At two sites, Año Nuevo and Monterey Bay, ancient otters had significantly higher δ^15^N values than modern populations. Amino acid δ^15^N data indicated this resulted from shifting baseline isotope values, rather than a change in sea otter trophic ecology. Our results help in better understanding the contemporary ecological role of sea otters and exemplify the strength of combing zooarchaeological and biological information to provide baseline data for conservation efforts.

## Graphical abstract

We measured the dietary niche of modern and ancient Southern sea otters in California using stable isotope analysis. We find the size of the realized niche in southern California has decreased over time. These results should be considered for effective management and conservation efforts of this threatened species.

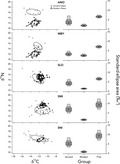

## 1 INTRODUCTION

**FIGURE 1:**
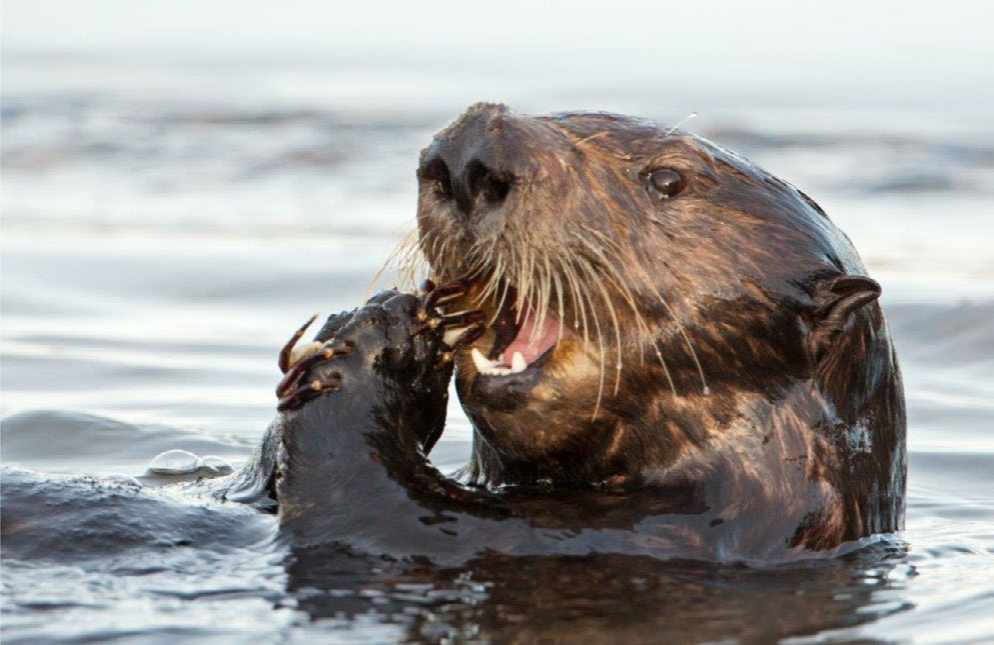
Southern sea otter (*Enhydra lutris nereis*) consuming a striped shore crab (*Pachygrapsus crassipes*). Photograph taken in Moss Landing, California, by Joe Tomoleoni. Used with permission

The sea otter, *Enhydra lutris* (Figure 1), is a marine mammal found in coastal nearshore ecosystems along the North Pacific. Across much of their range—Alaska, British Columbia, Washington, Oregon, and central California (Bodkin, 2015)—sea otters are considered a “keystone species” (Paine, 1969) disproportionately influencing ecosystem structure and function through indirect effects of predation (Estes & Palmisano, 1974; Hughes et al., 2016). The control by sea otters of key invertebrate populations prevents the overgrazing of kelp forests, and clears seagrass of harmful epiphytes, allowing these primary producers to flourish and provide habitat for a range of diverse taxa (Estes & Palmisano, 1974; Hughes et al., 2016; Steneck et al., 2002). Sea otters thus help maintain resilient coastal ecosystems, increase nearshore productivity, and provide valuable ecosystem services (Estes & Palmisano, 1974; Hughes et al., 2016; Wilmers, Estes, Edwards, Laidre, & Konar, 2012).

The current global sea otter population and range are severely reduced from historical levels, due to commercial fur trade activity in the 18th and 19th centuries (Riedman & Estes, 1990). Prior to the initiation of the North Pacific fur trade in the mid‐1700s, the global sea otter population may have been 150,000–300,000 individuals in a more or less contiguous distribution from Russia to Baja California (Riedman & Estes, 1990). Over the next ~150 years, sea otters were hunted nearly to extinction; by the early 20th century only 1,000–2,000 were left (Riedman & Estes, 1990). In 1911, when the International Fur Seal Treaty afforded sea otters some protection small remnant populations persisted in Russia, southwest Alaska, Haida Gwaii, Prince William Sound, and central California. From the mid‐1960s onwards, otters were periodically translocated from these remnant populations to southeastern Alaska, British Columbia, Washington, Oregon, and other California localities (Bodkin, 2015). Today, sea otter populations from eastern Russia to southeastern Alaska and British Columbia have recovered much of their prefur trade distribution (Riedman & Estes, 1990). In contrast, the coastal areas from Washington to southern California support relatively small and spatially isolated populations; this fragmented distribution reflects the failure of the Oregon translocation (Bodkin, 2015) and slow rate of recovery and natural range spread of the California population.

Despite the conservation success represented by postfur trade sea otter population recovery, it remains unclear whether sea otters have been fully restored to their historical ecological niches, which we here define as the combination of habitat (e.g., kelp forest versus soft sediment), and prey species utilized. We propose that industrial‐scale exploitation of the marine environment by humans (McCauley et al. 2015) may have led to a constriction of the ecological niche of modern sea otters relative to the past. This should be particularly true for the southern subspecies (*Enhydra lutris nereis*, Figure 1) which is currently found only in central California, an area with high human coastal densities and large‐scale fisheries. Sea otter recovery in this region has been sluggish, averaging only ~2% annual population growth (Tinker & Hatfield, 2018). Reasons suggested include the following: (a) the linear and narrow coastal shelf of California that limits access to unoccupied habitats (Lafferty & Tinker, 2014), (b) conflicts between otters and macroinvertebrate fisheries (Carswell, Speckman, & Gill, 2015), and (c) novel threats such as infectious disease and mortality caused by white sharks (Bodkin, 2015; Tinker & Hatfield, 2018; Tinker, Hatfield, Harris, & Ames, 2016). Defining a preindustrial ecological baseline for southern sea otters will thus benefit conservation efforts by identifying critical resources or habitats to protect, as well as potential functional roles and species interactions.

It is possible to develop a historical ecological baseline for sea otters because their bones are common in coastal archaeological sites (Jones, Culleton, Larson, Mellinger, & Porcasi, 2011; Misarti, Finney, Maschner, & Wooller, 2009; Szpak, Orchard, McKechnie, & Gröcke, 2012) and isotope‐based proxies allow for direct comparison of ancient and modern dietary niche and, indirectly, habitat. In sea otters, the isotopic niche is an established proxy for dietary niche, as otters consume a wide variety of macroinvertebrate prey fueled by two isotopically distinct sources of primary production: phytoplankton and macroalgae (Newsome et al., 2009; Page, Reed, Brzezinski, Melack, & Dugan, 2008). Consequently, bulk analysis of vibrissae or other tissues that record dietary inputs over long time scales provides an accurate and high‐resolution proxy for actual dietary niche breadth and variation, at both individual and population levels (Elliott Smith, Newsome, Estes, & Tinker, 2015; Newsome et al., 2009, 2015). Further, cutting‐edge isotopic techniques for analyzing individual amino acids within proteinaceous tissues can identify whether spatiotemporal shifts in bulk tissue isotope values are due to trophic level or baseline ecosystem changes (Chikaraishi et al., 2014; Whiteman, Elliott Smith, Besser, & Newsome, 2019). We can thus characterize the ecological niche of southern sea otter populations before and after their near extirpation by using bulk and amino acid isotope analysis of ancient and modern sea otter tissues.

Here, we use zooarchaeological collections and modern tissue samples collected from southern sea otters to (a) establish an ecological baseline for the species in California and (b) evaluate changes in their dietary niche over the past 7,000 years. We test whether the isotopic niche, measured from bulk tissue δ^13^C and δ^15^N values, occupied by sea otters has remained constant over time at five regions along the central and southern California coastline. We evaluate the extent that modern sea otters occupy the ancient isotopic space as a proxy for the recovery of their historical ecological niche. We also compare these data to the modern isotopic prey space. Finally, we use individual amino acid δ^15^N analysis to examine whether prey choice, environmental conditions (or both) have changed. Our results provide a framework for interpreting the contemporary ecological role of sea otters and for identifying potential areas of their historical niche that are underutilized and could be promoted with conservation efforts.

## 2 MATERIALS AND METHODS

### 2.1 Modern samples

**FIGURE 2:**
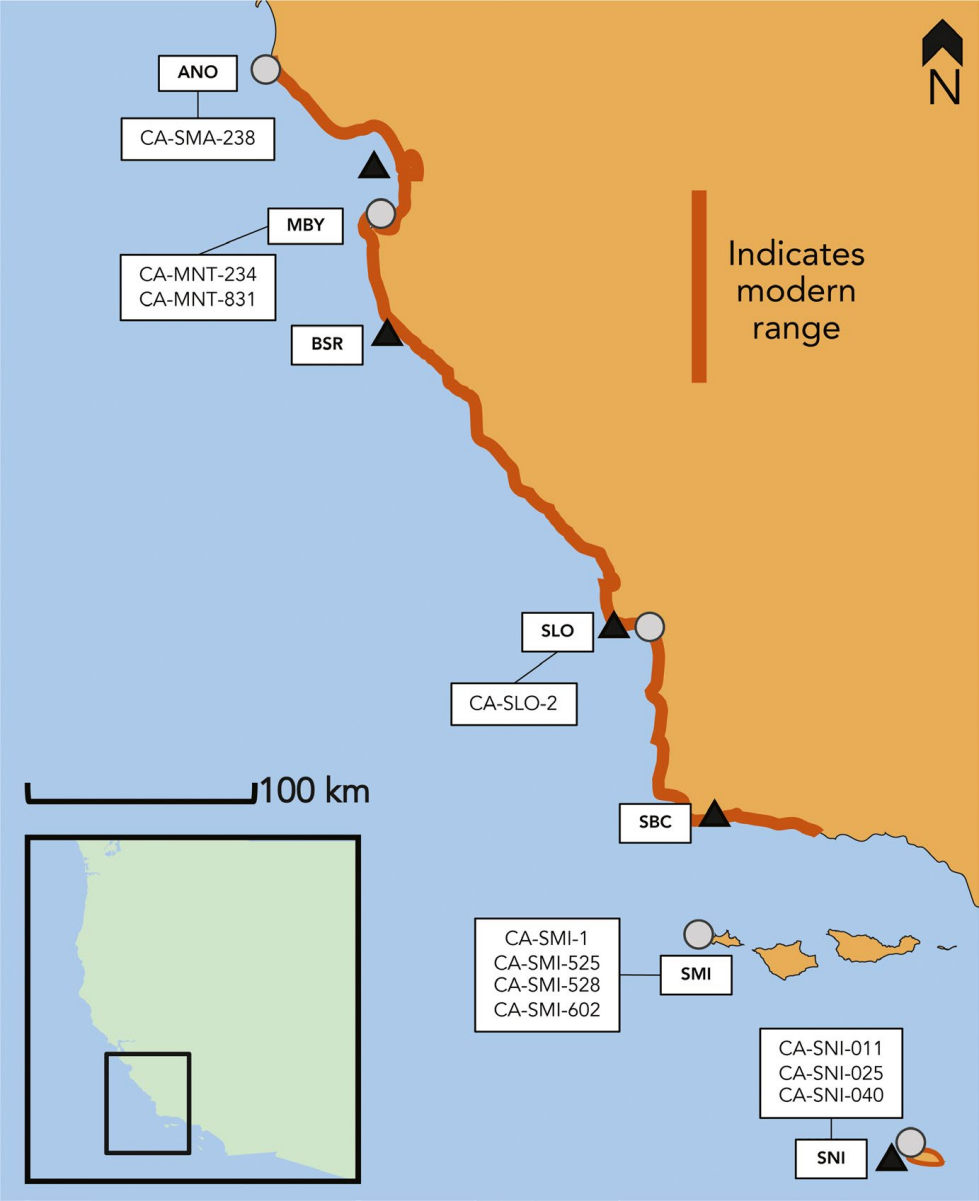
Locations of ancient and modern sea otter populations. Regions with archaeological southern sea otter remains represented by gray circles. Modern sea otter populations sampled for this study represented by black triangles (Elliott Smith et al., 2015). Modern range redrawn from Tinker and Hatfield (2018). Acronyms for regions are as follows: ANO, Año Nuevo; MBY, Monterey Bay; BSR, Big Sur Reserve; SLO, San Louis Obispo; SBC, Santa Barbara Channel; SMI, San Miguel Island; and SNI, San Nicolas Island. For a full list of sites and sample sizes, see Table 1

To characterize the modern isotopic niche, we used previously published isotopic data from vibrissae of 158 individuals in five subpopulations in California (Elliott Smith et al., 2015): Monterey Bay, Big Sur Reserve, San Louis Obispo, Santa Barbara Channel, and San Nicolas Island (Figure 2; Table 1). We sampled vibrissae from an additional seven San Louis Obispo individuals captured after 2015. Additional information on sample collection and identification can be found under Dryad accession https://doi.org/10.5061/dryad.ttdz08ktj. Our data encompass nearly the entire contemporary range of southern sea otters (Tinker & Hatfield, 2018) and represent a mix of males and females (Elliott Smith et al., 2015). All sampled individuals were independent foragers (weaned immature otters to aged adults), thus excluding dependent pups. To quantify the potential isotopic niche available for modern sea otters, we used published isotopic data from Newsome et al. (2015) on the prey types most commonly consumed in Monterey Bay and Big Sur (20 species) and San Nicolas Island (11 species) to define a possible isotopic prey space.

**TABLE 1:** Ancient and modern sea otter samples. For archaeological data, the age column represents years before present (yBP), based on calibrated AMS radiocarbon dates from the listed references. For modern samples, this represents the years of sea otter captures/collections (AD). With the exception of CA‐SLO‐2, all archaeological samples presented were analyzed in the current study. Modern sea otter data come from Elliott Smith et al., 2015; we analyzed an additional seven modern individuals from the San Louis Obispo region

Age	Site	Region	*N*	Age (cal yBP)	Reference
Ancient	CA‐SMA‐238	ANO	12	650–350	Hylkema (1991, 2019) Gifford‐Gonzalez (2011)
	CA‐MNT‐234	MBY	16	2,100–1,600	Gifford‐Gonzalez (2007)
	CA‐MNT‐831	MBY	6^a^	4,000–600 (Depending on strata)	Breschini & Haversat (2011)
	CA‐SLO‐2	SLO	24	7,000–300 (Depending on strata)	Jones et al. (2011)
	CA‐SMI‐1	SMI	8	7,000–3,400	Erlandson (1991)
	CA‐SMI‐525	SMI	12	3,100–500	Erlandson et al. (2005)
	CA‐SMI‐528 Strata 1	SMI	11	1,450–1,200	Walker, Kennett, Jones, & DeLong (2002)
	CA‐SMI‐602	SMI	3	500–300	Walker et al. (2002)
	CA‐SNI‐011	SNI	5	7,000–510 (Depending on strata)	Rick, Erlandson, Vellanoweth, & Braje (2005)
	CA‐SNI‐025	SNI	8	740–510	Martz (2008)
	CA‐SNI‐040	SNI	8	4,200–3,800	Ainis, Vellanoweth, Lapeña, & Thornber (2014)
Modern	Monterey Bay	MBY	31	AD 2000–2012	Elliott Smith et al. (2015)
	Big Sur Reserve	BSR	28	AD 2008–2012	Elliott Smith et al. (2015)
	San Louis Obispo	SLO	56	AD 2010–2013	Elliott Smith et al. (2015)
	Santa Barbara Channel	SBC	37	AD 2010–2013	Elliott Smith et al. (2015)
	San Nicolas Island	SNI	13	AD 2004	Elliott Smith et al. (2015)

Note

Acronyms for regions are as follows: ANO, Año Nuevo; MBY, Monterey Bay; BSR, Big Sur Reserve; SLO, San Louis Obispo; SBC, Santa Barbara Channel; SMI, San Miguel Island; and SNI, San Nicolas Island.

^a^ For MNT‐831, we were unable to confidently quantify the number of individuals due to poorly constrained sample ages and we thus excluded these data from some analyses (see Methods).

### 2.2 Archaeological samples

To characterize the historical isotopic niche, we sampled 107 bones from 10 California archaeological sites in close proximity to where modern sea otters were captured. These included both mainland and island archaeological sites and covered the entirety of the modern southern sea otter range—from Point Año Nuevo to San Nicolas Island (Table 1, Figure 2). We also include published isotope data from CA‐SLO‐2 near San Luis Obispo (Jones et al., 2011). Sample sizes and estimated site ages based on AMS radiocarbon dates are presented in Table 1; details on the excavation and identification of faunal remains can be found in the references therein. Care was taken not to resample individuals by considering specimen context (e.g., unit/level) and element. Within units/levels or for single component sites (e.g., SMA‐238), specimens were considered unique if they exhibited distinct (>1.0‰) δ^13^C or δ^15^N values (Clark, Horstmann, & Misarti, 2017). Where possible we sampled only adult or subadult individuals. From each specimen, we removed ~100‐mg of bone for stable isotope analysis using a Dremel tool.

### 2.3 Comparative tissue dataset

Comparison of modern and ancient sea otter samples necessitates isotope data from two distinct proteinaceous tissues: bone collagen and vibrissae keratin. These proteins can exhibit systematic isotopic differences commonly referred to as tissue‐specific isotope discrimination (Vanderklift & Ponsard, 2003). To quantify and correct for this, we sampled bone collagen, muscle, liver, and vibrissae from 29 sea otters stranded in central California from 2007 to 2014 (Additional information under Dryad accession https://doi.org/10.5061/dryad.ttdz08ktj). Tissues from stranded sea otter carcasses were collected through the California Sea Otter Stranding Network, a multi‐agency program coordinated by the California Department of Fish and Wildlife (CDFW) and USGS. Vibrissae were dry stored at 20°C, while liver and muscle were stored at −20°C; skulls were curated at the California Academy of Sciences. We sampled ~100 mg from all tissues.

### 2.4 Sample selection for amino acid δ^15^N (AA δ^15^N)

The isotopic analysis of individual amino acids (AA) within proteinaceous tissues is a cutting‐edge technique in ecological studies. By breaking down whole protein complexes and applying fundamental biochemical principles, it is possible to disentangle shifts in animals’ trophic ecology from ecosystem‐level changes associated with changes in the baseline isotopic composition of food webs (Chikaraishi et al., 2014). The δ^15^N of certain “source” amino acids (e.g., phenylalanine and lysine) are not heavily modified by animals during assimilation and tissue synthesis due to their lack of participation in metabolic processes such as deamination. Consequently, source amino acids experience little isotopic alteration as they move through food webs, providing an indicator of the baseline δ^15^N composition. Conversely, “trophic” amino acids, such as glutamic acid and proline, are heavily involved in metabolic processes and exhibit consistent ^15^N enrichment with each trophic step. Thus, the magnitude of the nitrogen isotope difference between source and trophic amino acids can be used as a proxy for the trophic level of an individual, whereas source amino acids can be used to infer baseline ecosystem δ^15^N composition (Chikaraishi et al., 2014; Whiteman et al., 2019).

To examine whether spatiotemporal changes in bulk tissue isotope values of sea otters were due to baseline isotopic or dietary shifts, we selected a subsample of 4–5 individuals from each archaeological region (excluding CA‐SLO‐2) for AA δ^15^N analysis (Table 3, Appendix S5). In addition, we selected five modern Monterey Bay individuals from stranded otter bone collagen samples (Table 3, Appendix S5). We did not analyze modern sea otter vibrissae for AA δ^15^N analysis because to our knowledge no study has addressed AA‐specific tissue discrimination.

### 2.5 Isotopic analysis

For both bulk analysis and amino acid analysis, we report all isotopic results as δ values: δ^13^C or δ^15^N = 1,000*[(R_samp_/R_std_) − 1], where R_samp_ and R_std_ are the ^13^C:^12^C or ^15^N:^14^N ratios of the sample and standard, respectively. Prior to analysis, bone collagen subsamples were cleaned of sediment and then demineralized with 0.25 N hydrochloric acid for 15–72 hr at 5°C. Each sample was then lipid extracted with three sequential 24 hr soaks in 2:1 chloroform:methanol and lyophilized after a deionized water rinse. Muscle and liver samples were also lipid extracted, rinsed, and lyophilized. Sea otter vibrissae were cleaned with 2:1 chloroform:methanol to remove surface contaminants. For bone, muscle, and liver, 0.5–0.6 mg of each subsample was weighed into 3 × 5 mm tin capsules. Vibrissae were subsampled following Newsome et al. (2009). For AA δ^15^N analysis, ~5–10 mg of extracted collagen was chemically processed following established protocols (Whiteman et al., 2019). See Appendix S1 for details on all isotopic measurements and quality control.

### 2.6 Data corrections and statistical analysis

We corrected for both tissue‐specific discrimination and temporal (Suess Effect; Cullen, Rosenthal, & Falkowski, 2001) isotopic shifts prior to analysis (Appendix S2). The resulting dataset (Dryad accession https://doi.org/10.5061/dryad.ttdz08ktj) violated a number of important assumptions of ANOVA. We thus tested for differences among modern and ancient sea otter δ^13^C and δ^15^N isotope values at each site using the nonparametric Cramér test (Baringhaus & Franz, 2004). We also employed Kruskal–Wallis, and pairwise Wilcoxon signed‐rank comparisons with Bonferroni adjusted p‐values to examine differences for each isotope system between ancient and modern otters; we report pairwise comparisons in Dryad accession https://doi.org/10.5061/dryad.ttdz08ktj. We likewise compared δ^13^C and δ^15^N isotopic values of ancient otters across different archaeological sites and regions. Our amino acid δ^15^N dataset did not exhibit deviations from normality, thus we used one‐way ANOVA to evaluate for differences in source and trophic AA δ^15^N, as well as trophic‐source offset among sites. We calculated pairwise trophic‐source offsets, as well as average offsets following methods from Bradley et al. (2015), using glutamic acid (Glu) and hydroxyproline/proline (Hyp‐Pro) as trophic AAs and phenylalanine (Phe) and lysine (Lys) as source AAs.

To characterize isotopic/dietary niche space and variability of ancient and modern otter populations, we used Bayesian standard ellipse areas (SEA_B_) (Stable Isotope Bayesian Ellipses in R; SIBER—Jackson, Inger, Parnell, & Bearhop, 2011). We ran the model with archaeological sites lumped within regions, and then with each site considered separately. In the latter case, we excluded the three samples from SMI‐602, and all samples from MNT‐831 which had poorly constrained age ranges. We also calculated SEA_B_ for modern California prey items from Monterey Bay/Big Sur and San Nicolas Island. We calculated median SEA_B_ and associated credibility intervals (SD) from 10,000 iterations of the model, as well as the proportion of SEA_B_ iterations from one group larger than the SEA_B_ of another group. Finally, we tested for the influence of time averaging on isotopic space (SEA_B_) using a linear model of median SEA_B_ versus time span occupied by each site (see Table 1 and Figure 5); modern samples were given a time span of 10 years. We performed all data corrections and statistical analyses using Program R (v.3.2.0).

## 3 RESULTS

### 3.1 Bulk δ^13^C and δ^15^N values

**FIGURE 3:**
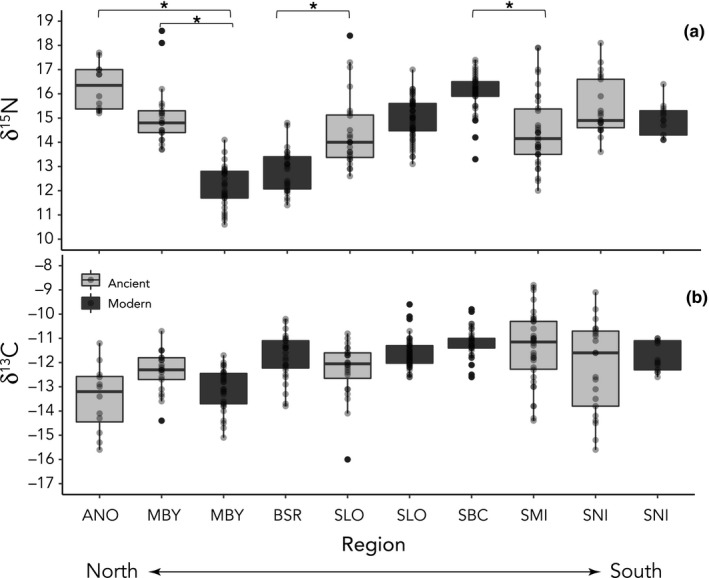
Boxplots of δ^15^N (panel A) and δ^13^C (panel B) values of modern and ancient sea otters. Gray boxes are ancient populations; black are modern. Bars with asterisks represent *ancient–modern* pairs that are statistically different at an adjusted *p* < .05 (see Table S4). Acronyms for regions are as follows: ANO, Año Nuevo; MBY, Monterey Bay; BSR, Big Sur Reserve; SLO, San Louis Obispo; SBC, Santa Barbara Channel; SMI, San Miguel Island; and SNI, San Nicolas Island. For a full list of sites and sample sizes, see Table 1

We found ancient and modern otters exhibited different multivariate distributions in all regions (Cramer's test: ANO/MBY/BSR *p* = .00; SLO *p* = .00; SMI/SBC *p* = .00; SNI *p* = .04). Univariate Kruskal–Wallis showed that ancient and modern otters had distinct δ^15^N values (H(9) = 159.1, *p* < .001) at almost every location (Dryad accession https://doi.org/10.5061/dryad.ttdz08ktj). Individual pairwise comparisons found ancient sea otter δ^15^N values from Año Nuevo and Monterey Bay were significantly higher than associated modern populations (Table S4, Figure 3). In contrast, ancient San Miguel Island otters had lower δ^15^N values than the modern Santa Barbara Channel population (Figure 3, Dryad accession https://doi.org/10.5061/dryad.ttdz08ktj). There were no significant differences in δ^13^C values between ancient and modern otters within regions (Figure 2, Appendix S6, Dryad accession https://doi.org/10.5061/dryad.ttdz08ktj). Among the modern sea otter populations, bulk δ^15^N varied with latitude: Modern Monterey Bay and Big Sur had lower average δ^15^N values in comparison with all other modern localities (Figure 3, Dryad accession https://doi.org/10.5061/dryad.ttdz08ktj).

### 3.2 Amino acid δ^15^N values

We found regional and temporal differences among otter populations in both source and trophic amino acids (Table 3, Appendix S5). One‐way ANOVA found significant differences in δ^15^N values between regions for the two commonly measured source ([Phe; *F*(4,18) = 5.5, *p* = .01]; [Lys; *F*(4,17) = 3.0, *p* = .03]), and trophic amino acids ([Glu; *F*(4,18) = 5.1, *p* = .01]; [Hyp‐Pro; *F*(4,8) = 6.6, *p* = .00]); data are presented in Table 3 and Appendix S5. However, we found no statistical difference in the average offset between trophic and source amino acids between ancient and modern otters [*F*(1,20) = 0.29, *p* = .60].

### 3.3 Isotopic niche breadth

Ancient sea otter populations occupied larger isotopic niches than their modern counterparts at almost every region. When archaeological sites within regions were combined, ancient San Louis Obispo, San Miguel Island, and San Nicolas Island populations exhibited larger standard ellipse areas (SEA_B_) than all other populations (Table 2, Appendix S3). When ancient data were grouped by archaeological site (excluding SMI‐602 and MNT‐831), results were very similar (Appendix S4). We also found differences in SEA_B_ of ancient and modern sea otter populations relative to the potential prey space produced by analysis of modern prey items. All modern sea otter populations occupied <35% of the median potential prey space, in stark contrast to archaeological sites that ranged from 31% (SMA‐238) to 99% (SNI‐011) of the modern potential prey space (Appendices S3 and S4).

**TABLE 2:** Isotopic standard ellipse areas for ancient and modern otters and potential prey. Median SIBER results are Bayesian metrics (SEA_B_) with associated 95% credibility intervals

b	Region	Type	Median (‰^2^)
Ancient	ANO	Otter	4.1 [2.5, 7.9]
	MBY	Otter	3.0 [2.0, 4.7]
	SLO	Otter	4.4 [3.0, 6.8]
	SMI	Otter	7.1 [5.1, 10.0]
	SNI	Otter	5.6 [3.7, 8.9]
Modern	MBY	Otter	2.1 [1.5, 3.1]
	BSR	Otter	2.6 [1.8, 3.8]
	SLO	Otter	1.6 [1.2, 2.0]
	SBC	Otter	1.5 [1.1, 2.1]
	SNI	Otter	1.0 [0.6, 1.8]
	MBY/BSR	Prey	6.8 [6.0, 7.7]
	SNI	Prey	9.6 [7.3, 12.9]

Note

Acronyms for regions are as follows: ANO, Ano Nuevo; MBY, Monterey Bay; BSR, Big Sur Reserve; SLO, San Louis Obispo; SBC, Santa Barbara Channel; SMI, San Miguel Island; and SNI, San Nicolas Island.

To evaluate the effect of time averaging, we compared the median SEA_B_ of all ancient and modern sites and modern prey (excluding SMI‐602 and MNT‐831) to the estimated time span represented by each site (Table 1). A resulting linear model found no effect of time span on ellipse area size (R^2^ = 0.06, *p* = .19; Figure 5), indicating differences in ellipse area between ancient and modern populations are driven by biological patterns and not statistical artifacts.

## 4 DISCUSSION

Our results provide evidence of a reduced dietary (isotopic) niche of southern sea otters in portions of their current range. We find that the majority of sampled ancient otter populations in California, ranging in age from 7,000 to 350 years before present, had a wider dietary niche than their modern counterparts. In particular, ancient otters from San Miguel and San Nicolas Islands had the largest dietary niche space of all populations measured. This suggests that the current high diversity of invertebrate prey communities south of Point Conception relative to central California (Graham, Halpern, & Carr, 2008) has played a role in the past. We also find differences among ancient and modern sea otter populations in the relationship between average δ^15^N values and latitude, which is likely driven by a combination of oceanographic factors (baseline) and diet composition (trophic level). Our work provides important context for understanding modern sea otter dietary patterns and allows for predictions of how their ecology will change in the future.

### 4.1 Isotopic niche of ancient and modern sea otters

**FIGURE 4:**
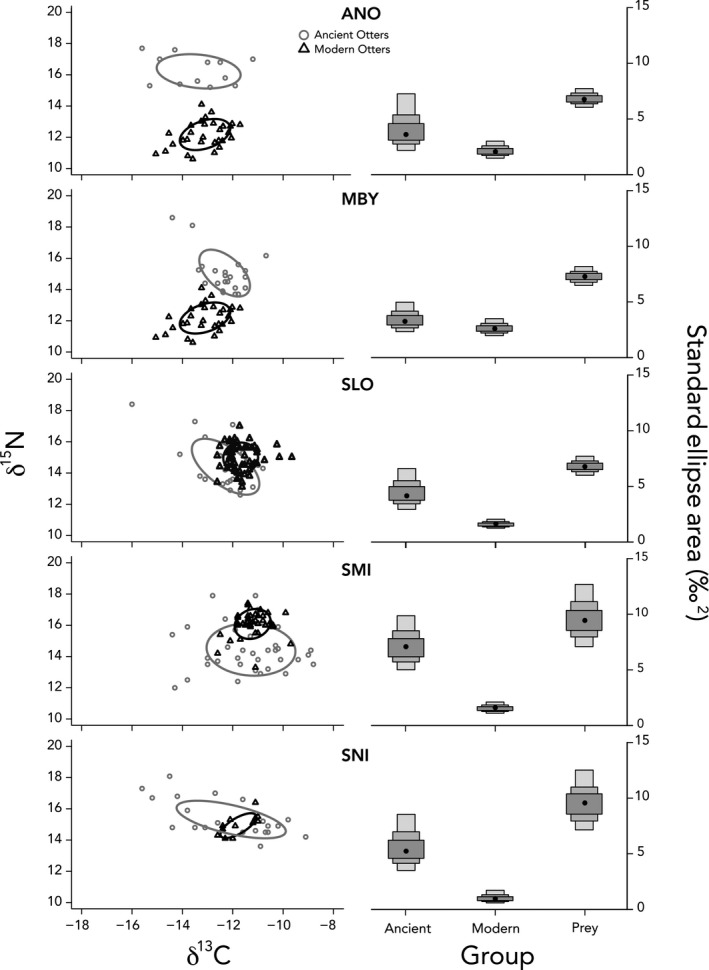
Isotopic niche space of ancient and modern sea otter populations. Left‐side panels show δ^13^C and δ^15^N values of ancient (gray circles) and modern (black diamonds) sea otters. Ellipses represent 50% of the total amount of isotopic space occupied by each population. Right‐side shows median Bayesian ellipse size ± credibility intervals for each population. All sea otter data were also compared to modern prey data from Newsome et al. (2015). From top to bottom, the ancient/modern comparisons are as follows: ancient Año Nuevo otters (ANO) with modern Monterey Bay otter and prey (MBY), ancient MBY with modern MBY and prey, ancient San Louis Obispo (SLO) with modern SLO and MBY prey, ancient San Miguel Island (SMI) with modern Santa Barbara Channel (SBC) and San Nicolas Island prey, and ancient San Nicolas Island (SNI) with modern SNI and prey. For a full list of sites and sample sizes, see Table 1

We found striking differences in the isotopic niche space of ancient and modern sea otters in California, particularly in the southern portion of the range. Ancient otters from the five regions occupied a larger isotopic niche than nearly all modern localities (Figure 4, Table 2, Appendix S3). However, at the three most southerly sites, ancient otters exhibited SEA_B_ more than *twice* as large as modern counterparts (Figure 4, Table 2, Appendix S3). We suspect this is due to the diversity of nearshore marine communities in southern California. The area near the Santa Barbara Channel is a major zoogeographic transition zone (Figure 2), with nearshore and island ecosystems south of Point Conception having a greater complexity of substrate, milder wave environments, and more variable upwelling regimes in comparison with the rest of California (Graham et al., 2008). As a result, these areas support a greater diversity of invertebrate and vertebrate taxa, and higher rates of endemism than sites in central and northern California (Graham et al., 2008; Seapy & Littler, 1980). This plethora of secondary prey species likely contributed to the greater dietary diversity of ancient sea otter populations in this region. In particular, we note the striking variability in δ^13^C values among ancient otters from San Miguel and San Nicolas Islands (−14‰ to −9‰) in comparison with modern counterparts (−12‰ to −10‰; Figure 4). These data suggest that ancient populations from these regions foraged in both rocky, kelp‐dominated habitats, and soft‐sediment, phytoplankton‐fueled habitats, characterized by high and low δ^13^C values, respectively (Page et al. 2008). The use of soft‐sediment habitats by otters is now recognized as an important aspect of their ecology (e.g., Hughes et al. 2016), and our results are consistent with a greater historical reliance on these systems in ancient southern California.

Despite this larger potential isotopic niche, modern sea otters in southern California exhibit low degrees of dietary (isotopic) variation, a pattern we suggest is a function of the time since recolonization and low population densities. Southern sea otter populations at San Nicolas Island and the Santa Barbara Channel are at low densities in comparison with the more northerly sites of Monterey Bay and Big Sur (Tinker, Bentall, & Estes, 2008, 2019); the 12 individuals analyzed here from San Nicolas represented nearly a third of the population around the island at the time (Tinker et al., 2008, Elliott Smith et al.^2015^). Decades of observational studies document that recently established sea otter populations at low density target the largest, most energy‐rich prey, leading to low levels of dietary diversity and a small population‐level isotopic niche space (Estes, Riedman, Staedler, Tinker, & Lyon, 2003; Tinker et al., 2008, 2012). As densities increase, high‐ranked resources become depleted, and individuals broaden their diet resulting in an increase in population‐level dietary/isotopic niche breadth (Newsome et al., 2009; Tinker et al., 2008). This latter pattern can be seen in modern sea otters in the northerly sites of Monterey Bay and Big Sur, where otters are likely near carrying capacity (Tinker et al., 2019). Importantly, in these areas, the size of the ancient sea otter isotopic niche is similar to their modern counterparts, indicating populations in central California have largely recovered their historical dietary breadth (Figure 4, Table 2, Appendix S3). In contrast, the isotopic data from archaeological remains of sea otters in southern California suggest that populations in this region could occupy a much larger dietary niche than currently observed.

### 4.2 Latitudinal trends in modern and ancient sea otter isotopic values

Among modern otters, there was a ~4‰ increase in mean δ^15^N with decreasing latitude (Figure 3). Some of this can be explained by a ~1–2‰ increase in baseline δ^15^N values along this section of coastline, assumed to be driven by northward flow and upwelling of ^15^N‐enriched intermediate waters of the California Undercurrent (Vokhshoori & McCarthy, 2014). However, the majority of the increase in modern δ^15^N values likely results from increased consumption of upper trophic level invertebrates by sea otters in the Santa Barbara Channel and San Nicolas Island. Behavioral observations suggest otters in these localities have a preference for urchins, large carnivorous crabs, and octopus (USGS, unpublished data; Tinker et al., 2008), which would explain the relatively high δ^15^N values of sea otters at southern sites (Figures 2 and 3; Newsome et al., 2009). In contrast, high densities of otters in Monterey Bay and Big Sur mean they include a greater amount of smaller, lower trophic level invertebrates in the diet (Tinker et al., 2008) and thus have lower δ^15^N values (Newsome et al., 2009).

Among ancient otter populations, we found no overall trend with decreasing latitude; however, we did find differences in mean δ^15^N between ancient and modern otters at some sites. Ancient otters from Año Nuevo and Monterey Bay had significantly higher δ^15^N values than modern counterparts (Figure 3, Dryad accession https://doi.org/10.5061/dryad.ttdz08ktj). Given the time averaging inherent in ancient data—some localities representing >2,000 years—we suspect the density‐dependent processes driving the consumption of predominantly high trophic level prey is not likely to be the causal factor. Instead, we suspect a change in the underlying baseline isotope values of the northern regions (Ruiz‐Cooley, Koch, Fiedler, & McCarthy, 2014). We tested for this by comparing amino acid δ^15^N data of a subsample of ancient otters from Año Nuevo, Monterey Bay, San Miguel Island, and San Nicolas Island, to modern otters from Monterey Bay (Table 3, Appendix S5).

**TABLE 3:** δ^15^N values of individual amino acids from a subset of ancient and modern sea otters. Presented here are two commonly reported “trophic” and “source” amino acids, respectively, glutamic acid (Glu), hydroxyproline‐proline (Hyp‐Pro), and phenylalanine (Phe), and lysine (Lys). Also reported is the mean (±SD) Glu‐Phe offset for each locale and the average offset between these trophic and source amino acids as calculated following methods by Bradley et al. (2015).

Age	Locale	Glu δ^15^N	Hyp‐Pro δ^15^N	Phe δ^15^N	Lys δ^15^N	Glu‐Phe Offset	Average Trophic‐source offset
Ancient otters	ANO	24.0 ± 1.6	22.2 ± 1.6	12.3 ± 1.1	10.5 ± 0.7	11.6 ± 2.5	11.7 ± 1.8
	MBY	21.9 ± 1.6	19.4 ± 1.8	10.7 ± 0.5	9.1 ± 1.0	11.2 ± 1.7	9.9 ± 2.0
	SMI	20.2 ± 0.9	18.4 ± 0.4	9.8 ± 0.9	9.2 ± 0.8	10.5 ± 1.3	9.2 ± 1.2
	SNI	21.0 ± 0.2	20.0 ± 0.8	12.0 ± 0.9	11.2 ± 0.8	9.0 ± 1.1	8.3 ± 1.8
Modern otters	MBY	22.9 ± 1.7	20.8 ± 0.4	10.3 ± 1.4	9.6 ± 1.2	12.6 ± 1.4	11.7 ± 1.6

Acronyms for regions are as follows: ANO, Año Nuevo; MBY, Monterey Bay; SMI, San Miguel Island; and SNI, San Nicolas Island. See Appendix S5 for δ^15^N data of all individual amino acids measured.

Amino acid δ^15^N analysis indicated that differences in modern and ancient sea otter bulk values (e.g., Figures 2 and 3) are due to a shifting isotopic baseline in the California Current System over the Holocene. Notably, we did not find a significant difference in the average offset between our trophic and source amino acid δ^15^N values between locales or time periods (Table 3), which indicates that the trophic level of sea otters has not significantly changed over the past 7,000 years (Chikaraishi et al., 2014; Whiteman et al., 2019). Instead, we found differences in the baseline δ^15^N values among regions with northerly ancient Año Nuevo (12.3 ± 1.1‰) otters exhibiting the highest mean (±SD) Phe (source) δ^15^N values, and southerly ancient San Miguel Island otters the lowest (9.8 ± 0.9‰; Table 3), a pattern opposite to that observed today (Vokhshoori & McCarthy, 2014). In addition, we noted a wide range in the mean (±SD) differences between otter trophic and source δ^15^N values between sites, with ancient San Nicolas Island otters having the lowest (8.3 ± 1.8‰), and modern Monterey Bay (11.7 ± 1.6‰) and ancient Año Nuevo (11.7 ± 1.8‰) the highest, offsets (Table 3). This variation likely reflects the trophic flexibility of otters as documented in the modern through high‐resolution observational datasets (Estes et al., 2003; Tinker et al., 2008).

### 4.3 Zooarchaeological data and modern southern sea otter ecology

**FIGURE 5:**
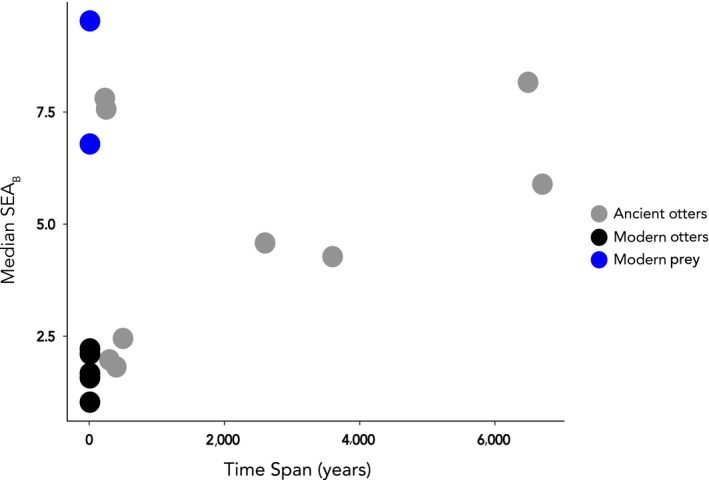
Relationship between median SEA_B_ and time span. Time span for each archaeological site presented in Table 1; modern prey and otters were given a time span of 10 years

The analysis of ancient faunal remains from archaeological sites poses a number of quantitative and theoretical issues when comparing them to modern ecological datasets. Most notably, archaeological sites in even the best scenarios represent time‐averaged assemblages on the order of hundreds to thousands of years, and so can never realize the high resolution of modern ecological sampling conducted over seasonal to decadal timescales (Rick & Lockwood, 2013). Here, our ancient sea otter samples are likely an aggregation of multiple generations. In addition, we cannot rule out the modification of local environments by humans. However, our use of zooarchaeological collections is biologically relevant for two reasons. First, we found no relationship between the isotopic niche space (SEA_B_) of ancient otters versus the amount of time represented by each archaeological or modern population (Figure 5), indicating that results are driven by real biological patterns and not a statistical artifact. Second, when characterizing the historical ecological niche, incorporation of data across hundreds or even thousands of years provides a more representative view of a species ecology than a seasonal or multi‐annual snapshot. Such an approach, which examines ecological patterns over evolutionarily relevant timescales, is a proven way of establishing conservation baselines and characterizing the plasticity of animals to long‐term natural or anthropogenic environmental change (e.g., Jackson et al., 2001; Rick & Lockwood, 2013).

Our results have implications for the conservation of southern sea otters, and for efforts to minimize negative interactions between otters and human populations. Our findings suggest sea otters have a much greater potential niche space in southern California than currently occupied and further, that this niche may be larger than that utilized by modern, high density, sea otter populations in central California (Tinker et al., 2008, 2019). Southern sea otters are currently expanding their range into southern California where they have not occurred for centuries; our data provide clues as to how their diets will broaden as they continue to establish south of Point Conception. However, the ancient dietary niche space of southern sea otters that we have characterized here was prior to the development of commercial fisheries. Over the past two centuries, intensive fishing has exploited several of the main macroinvertebrate species consumed by sea otters, including abalone, and red urchins (Dayton, Tegner, Edwards, & Riser, 1998; Leet, 2001). Consequently, the historical dietary niche occupied by sea otters may be unobtainable until conservation and management efforts restore higher densities of important invertebrate prey. Despite this, our dataset speaks to a long‐term history of interactions between humans and sea otters. The coastal archaeological record (e.g., Jones et al., 2011; Misarti et al., 2009; Szpak et al., 2012) demonstrates that humans have been living with, and harvesting, sea otters and their prey items for at least 10,000 years. The isotopic data we present here show that despite this, ancient sea otters had an ecological niche equivalent to, or greater than modern populations, suggesting that they occurred at high density in the past despite being subjected to harvest pressure. Such insights may aid in developing species‐ or ecosystem‐based management plans that promote long‐term sustainable relationships between the competing needs of humans and top predators.

## CONFLICT OF INTEREST

None declared.

## AUTHOR CONTRIBUTION

EES and SDN formulated the research questions. ELW, DJK, RLV, DGG, and MGH excavated, identified, and assisted with the isotopic sampling of all zooarchaeological material. MTT provided assistance with obtaining modern comparative specimens. EES and SDN conducted isotopic analyses. EES and MTT conducted statistical analyses. EES wrote the manuscript with editorial advice from all coauthors.

## Supporting information

Supplementary Material

## Data Availability

All data used for this work are publicly available in Dryad accession https://doi.org/10.5061/dryad.ttdz08ktj
