# Supplementary material for: Reductions in the dietary niche of southern sea otters (Enhydra lutris nereis) from the Holocene to the Anthropocene
Source: Ecol Evol. 2020 Mar 10;10(7):3318–29. doi: 10.1002/ece3.6114 (PMC7141068; doi:10.1002/ece3.6114)
Supplement: Supplementary file 1 — Supplementary Material [file ECE3-10-3318-s001.docx]

**APPENDIX 1.**

**Sample preparation and analysis for bulk and amino acid δ^15^N.**

For both bulk and amino acid analysis we report all isotopic results as δ values: δ^13^C or δ^15^N = 1000*[(Rsamp/Rstd) $-$ 1], where Rsamp and Rstd are the ^13^C: ^12^C or ^15^N:^14^N ratios of the sample and standard, respectively. The internationally accepted standards for δ^13^C and δ^15^N analysis are Vienna-Pee Dee Belemnite limestone (V-PDB) and atmospheric N_2_. The units are expressed as parts per thousand, or per mil (‰). For bulk analysis, δ13C and 􏰀δ15N values of all samples were analyzed using a Costech 4010 elemental analyzer (Valencia, CA) interfaced with a Thermo Scientific Delta V Plus isotope ratio mass spectrometer (Bremen, Germany) at the University of New Mexico Center for Stable Isotopes (UNM-CSI, Albuquerque, NM). The standard deviation of organic reference materials within a run was $\leq$0.2‰ for both δ^13^C and δ^15^N values. As a control for the quality of our ancient collagen samples, we also measured [C]:[N] ratios. The theoretical weight percent [C]:[N] of unaltered bone collagen falls between 2.8-3.5 (Ambrose 1990), all but two samples fell within this threshold (Dryad accession <https://doi.org/10.5061/dryad.ttdz08ktj>). For all analyses using modern vibrissae, we used the mean δ13C and 􏰀δ15N values across all subsampled segments from an individual.

For AA δ^15^N analysis, ~5-10mg of extracted collagen was hydrolyzed to constituent amino acids in 1mL of 6N hydrochloric acid (HCl) at 110°C for 20 hours; tubes were flushed with N_2_ to prevent oxidation during hydrolysis. After hydrolysis, amino acids were derivatized to *N*- trifluoroacetic acid isopropyl esters following established protocols (Whiteman et al. 2019). Samples were derivatized in batches of 8–17 along with an in-house reference material containing all amino acids we measured. δ^15^N values of individual derivatized amino acids were measured using a GC-C-IRMS system at UNM-CSI. Derivatized samples were injected into a 60m BPx5 gas chromatograph column for amino acid separation (0.32 ID, 1.0μm film thickness, SGE Analytical Science, Victoria, Australia) in a Thermo Scientific Trace 1300, combusted into CO_2_ gas via a Thermo Scientific GC Isolink II, and analyzed with a Thermo Scientific Delta V Plus. Samples were run in duplicate or triplicate and bracketed with our reference material. AA data for a sample were accepted if the standard deviations was <1.0‰ across injections. The within-run standard deviations of measured δ^15^N values among AA in the in-house reference material ranged from 1.7‰ (Glycine) to 0.1‰ (Aspartic Acid). Due to peak coelution, we were unable to reliably distinguish between hydroxyproline (Hyp) and proline (Pro), thus these data are presented as a pooled isotope value (Hyp-Pro).

**References:**

Whiteman, J. P. et al. 2019. A guide to using compound-specific stable isotope analysis to study the fates of molecules in organisms and ecosystems. Diversity, 11, 8.

Ambrose, S. H. (1990). Preparation and characterization of bone and tooth collagen for isotopic analysis. Journal of Archaeological Science, 17, 431–451.

**APPENDIX 2.**

**Tissue specific and temporal isotope corrections.**

A one-way ANOVA of different tissues from stranded sea otters indicated systematic differences in δ13C between bone collagen and vibrissae: otter bone collagen had higher mean δ13C values by +0.9 ± 0.4‰ ([F (3, 112) = 160, p < 0.001]; Table S3). Thus, we added +1.0‰ to modern sea otter vibrissae δ13C values so these data could be directly compared to bone collagen. We found no significant difference in δ15N between bones and whiskers (Dryad accession <https://doi.org/10.5061/dryad.ttdz08ktj>). In addition, we applied a conservative correction of -1.5‰ to ancient sea otter δ13C values to account for the decrease in atmospheric CO_2_ δ13C over the past 150 years driven by fossil fuel combustion (i.e., the Suess effect; Cullen et al. 2001). There is no known temporal correction for δ15N.

**References**:

Cullen JT, Rosenthal Y, Falkowski PG. 2001. The effect of anthropogenic CO_2_ on the carbon isotope composition of marine phytoplankton. Limnology and Oceanography **46**:996–998.

**APPENDIX 3.**

**Comparison of isotopic niche width between ancient and modern sea otter populations.**

Each cell represents the proportion of draws (from 10,000 Bayesian iterations) from the row group that are ***larger*** than the column group. Acronyms for regions are as follows: ANO = Año Nuevo, MBY = Monterey Bay, BSR = Big Sur Reserve, SLO = San Louis Obispo, SBC = Santa Barbara Channel, SMI = San Miguel Island, SNI = San Nicolas Island. ‘mod’ = modern populations, ‘arc’ = ancient. Here, comparisons are population-wide: ancient MBY combines MNT-234 and MNT-831, ancient SMI combines SMI-1, SMI-525, SMI-528, and SMI-602. Ancient SNI combines SNI-011, SNI-025 and SNI-040. All samples are also compared to the isotopic niche width of modern potential prey items from Monterey Bay/Big Sur and San Nicolas Island (see Newsome et al. 2015).

|  |  | **Ancient Populations** | | | | | **Modern Populations** | | | | | **Modern Prey** | |
| --- | --- | --- | --- | --- | --- | --- | --- | --- | --- | --- | --- | --- | --- |
|  |  | **ANO** | **MBY** | **SLO** | **SMI** | **SNI** | **MBY** | **BSR** | **SLO** | **SBC** | **SNI** | **MBY/BSR** | **SNI** |
| **Ancient Populations** | **ANO** | – | 0.83 | 0.42 | 0.06 | 0.21 | 0.98 | 0.92 | 1.00 | 1.00 | 1.00 | 0.07 | 0.01 |
|  | **MBY** | – | – | 0.09 | 0.00 | 0.02 | 0.88 | 0.69 | 1.00 | 0.99 | 1.00 | 0.00 | 0.00 |
|  | **SLO** | – | – | – | 0.04 | 0.22 | 1.00 | 0.97 | 1.00 | 1.00 | 1.00 | 0.03 | 0.00 |
|  | **SMI** | – | – | – | – | 0.81 | 1.00 | 1.00 | 1.00 | 1.00 | 1.00 | 0.61 | 0.10 |
|  | **SNI** | – | – | – | – | – | 1.00 | 1.00 | 1.00 | 1.00 | 1.00 | 0.20 | 0.02 |
| **Modern Populations** | **MBY** | – | – | – | – | – | – | 0.25 | 0.92 | 0.92 | 0.98 | 0.00 | 0.00 |
|  | **BSR** | – | – | – | – | – | – | – | 0.99 | 0.98 | 1.00 | 0.00 | 0.00 |
|  | **SLO** | – | – | – | – | – | – | – | – | 0.56 | 0.91 | 0.00 | 0.00 |
|  | **SBC** | – | – | – | – | – | – | – | – | – | 0.89 | 0.00 | 0.00 |
|  | **SNI** | – | – | – | – | – | – | – | – | – | – | 0.00 | 0.00 |
| **Modern Prey** | **MBY/BSR** | – | – | – | – | – | – | – | – | – | – | – | 0.01 |
|  | **SNI** | – | – | – | – | – | – | – | – | – | – | – | – |

**APPENDIX 4.**

**Comparison of isotopic niche width between individual archaeological sites and modern sea otter populations.**

Each cell represents the proportion of draws (from 10,000 Bayesian iterations) from the row group that are ***larger*** than the column group. Archaeological sites are listed by name: SMI-602 and MNT-831 excluded because of small sample size and provenance concerns. Modern sites are listed by region. Acronyms for regions are as follows: ANO = Año Nuevo, MBY = Monterey Bay, BSR = Big Sur Reserve, SLO = San Louis Obispo, SBC = Santa Barbara Channel, SMI = San Miguel Island, SNI = San Nicolas Island. All samples are also compared to the isotopic niche width of modern potential prey items from Monterey Bay/Big Sur and San Nicolas Island (see Newsome et al. 2015).

|  |  | **Archaeological Sites** | | | | | | | | | **Modern Sites** | | | | | **Modern Prey** | |
| --- | --- | --- | --- | --- | --- | --- | --- | --- | --- | --- | --- | --- | --- | --- | --- | --- | --- |
|  |  | **SMA-238** | **MNT-234** | **SLO-2** | **SMI-1** | **SMI-525** | **SMI-528** | **SNI-011** | **SNI-025** | **SNI-040** | **MBY** | **BSR** | **SLO** | **SBC** | **SNI** | **MBY/BSR** | **SNI** |
| **Archaeological Sites** | **SMA-238** | – | 0.21 | 0.01 | 0.04 | 0.02 | 0.00 | 0.00 | 0.00 | 0.59 | 0.68 | 0.36 | 0.75 | 0.98 | 0.43 | 0.00 | 0.00 |
|  | **MNT-234** | – | – | 0.03 | 0.14 | 0.07 | 0.00 | 0.01 | 0.01 | 0.82 | 0.94 | 0.72 | 0.98 | 1.00 | 0.74 | 0.00 | 0.00 |
|  | **SLO-2** | – | – | – | 0.65 | 0.59 | 0.13 | 0.15 | 0.13 | 0.99 | 1.00 | 1.00 | 1.00 | 1.00 | 0.99 | 0.09 | 0.01 |
|  | **SMI-1** | – | – | – | – | 0.43 | 0.12 | 0.13 | 0.12 | 0.96 | 1.00 | 0.96 | 1.00 | 1.00 | 0.95 | 0.12 | 0.03 |
|  | **SMI-525** | – | – | – | – | – | 0.12 | 0.14 | 0.13 | 0.98 | 1.00 | 0.99 | 1.00 | 1.00 | 0.97 | 0.11 | 0.02 |
|  | **SMI-528** | – | – | – | – | – | – | 0.45 | 0.47 | 1.00 | 1.00 | 1.00 | 1.00 | 1.00 | 1.00 | 0.64 | 0.25 |
|  | **SNI-011** | – | – | – | – | – | – | – | 0.54 | 1.00 | 1.00 | 1.00 | 1.00 | 1.00 | 1.00 | 0.66 | 0.38 |
|  | **SNI-025** | – | – | – | – | – | – | – | – | 1.00 | 1.00 | 1.00 | 1.00 | 1.00 | 1.00 | 0.66 | 0.30 |
|  | **SNI-040** | – | – | – | – | – | – | – | – | – | 0.56 | 0.30 | 0.63 | 0.93 | 0.35 | 0.00 | 0.00 |
| **Modern Sites** | **MBY** | – | – | – | – | – | – | – | – | – | – | 0.15 | 0.61 | 0.98 | 0.25 | 0.00 | 0.00 |
|  | **BSR** | – | – | – | – | – | – | – | – | – | – | – | 0.93 | 1.00 | 0.57 | 0.00 | 0.00 |
|  | **SLO** | – | – | – | – | – | – | – | – | – | – | – | – | 0.97 | 0.17 | 0.00 | 0.00 |
|  | **SBC** | – | – | – | – | – | – | – | – | – | – | – | – | – | 0.01 | 0.00 | 0.00 |
|  | **SNI** | – | – | – | – | – | – | – | – | – | – | – | – | – | – | 0.00 | 0.00 |
| **Prey** | **MBY/BSR** | – | – | – | – | – | – | – | – | – | – | – | – | – | – | – | 0.01 |
|  | **SNI** | – | – | – | – | – | – | – | – | – | – | – | – | – | – | – | – |

**APPENDIX 5.**

**Amino Acid δ^15^N data of ancient and modern sea otters.**

Values represent averages ± SD across injections of a sample; see Appendix 1. Abbreviations for amino acids are as follows: alanine (Ala), glycine (Gly), serine (Ser), valine (Val), leucine (Leu), isoleucine (Ile), hydroxyproline/proline (Hyp-Pro), aspartic acid (Asp), glutamic acid (Glu), phenylalanine (Phe), lysine (Lys). ‘ND’ indicates we were not able to reliably measure the given amino acid. Modern samples are bone collagen from our stranded tissue dataset. Acronyms for Region are as follows: ANO = Año Nuevo, MBY = Monterey Bay, SMI = San Miguel Island, SNI = San Nicolas Island.

| **SampleID** | **Type** | **Region** | **Ala15N** | **Gly15N** | **Ser15N** | **Val15N** | **Leu15N** | **Ile15N** | **Hyp-Pro15N** | **Asp15N** | **Glu15N** | **Phe15N** | **Lys15N** |
| --- | --- | --- | --- | --- | --- | --- | --- | --- | --- | --- | --- | --- | --- |
| SO-SMA-146 | Ancient | ANO | 21.2 ± 0.7 | 11.7 ± 0.6 | 12.9 ± 0.4 | 23.1 ± 0.3 | 21.3 ± 0.7 | 20.1 ± 0.9 | 22.9 ± 0.4 | 20.7 ± 0.3 | 23.1 ± 0.5 | 13.3 ± 0.2 | 11.1 ± 0.5 |
| SO-SMA-168 | Ancient | ANO | 25.8 ± 0.2 | 14.3 ± 0.6 | 15.3 ± 0.5 | 24.8 ± 0.4 | 24.7 ± 0.4 | 23.5 ± 0.3 | 24.1 ± 0.3 | 22.2 ± 0.5 | 26.2 ± 0.6 | 12.3 ± 0.1 | 10.0 ± 0.3 |
| SO-SMA-252 | Ancient | ANO | 21.0 ± 0.5 | 12.3 ± 0.6 | 12.9 ± 0.3 | 22.1 ± 0.4 | 19.3 ± 0.1 | 20.0 ± 0.3 | 21.2 ± 0.2 | 19.3 ± 0.7 | 22.1 ± 0.4 | 13.1 ± 0.4 | 11.1 ± 0.6 |
| SO-SMA-739 | Ancient | ANO | 23.1 ± 0.1 | ND | 13.4 ± 0.6 | 23.8 ± 0.2 | 22.2 ±0.1 | 23.4 ± 0.6 | 20.1 ± 0.2 | 20.2 ± 0.5 | 23.4 ± 0.0 | 12.6 ± 0.2 | 10.8 ± 0.0 |
| SO-SMA-751 | Ancient | ANO | 25.8 ± 0.0 | 14.5 ± 0.0 | 13.5 ± 0.4 | 24.5 ± 0.4 | 23.3 ± 0.2 | 23.6 ± 0.9 | 22.6 ± 0.1 | 22.0 ± 0.2 | 25.1 ± 0.2 | 10.5 ± 0.8 | 9.5 ± 0.2 |
| SO-MNT-234-2724 | Ancient | MBY | 21.1 ± 0.1 | 12.0 ± 0.0 | 11.0 ± 0.3 | 21.4 ± 0.5 | 20.2 ± 0.1 | 21.4 ± 0.3 | 21.2 ± 0.1 | 19.8 ± 0.1 | 22.3 ± 0.1 | 9.4 ± 0.5 | 11.2 ± 0.3 |
| SO-MNT-234-2977 | Ancient | MBY | 23.0 ± 0.0 | 10.9 ± 0.2 | 12.0 ± 0.6 | 22.3 ± 0.3 | 22.5 ± 0.1 | 22.6 ± 0.7 | 20.7 ± 0.1 | 21.4 ± 0.1 | 23.9 ± 0.2 | 12.1 ± 0.1 | 10.1 ± 0.1 |
| SO-MNT-234-4825 | Ancient | MBY | 24.1 ± 0.6 | 12.4 ± 0.3 | 10.9 ± 0.9 | 23.8 ± 0.4 | 23.5 ± 0.4 | 23.6 ± 0.7 | 21.2 ± 0.2 | 21.7 ± 0.3 | 25.2 ± 0.2 | 11.1 ± 0.5 | 9.1 ± 0.0 |
| MNT-831-EL-005 | Ancient | MBY | ND | 10.3 ± 0.3 | 11.6 ± 0.3 | 20.6 ± 0.1 | 20.4 ± 0.6 | 20.9 ± 0.9 | 20.7 ± 0.0 | 19.7 ± 0.3 | 21.9 ± 0.5 | 8.6 ± 0.4 | 7.9 ± 0.1 |
| MNT-831-EL-018 | Ancient | MBY | ND | 14.3 ± 0.2 | 13.2 ± 0.3 | 21.7 ± 0.0 | 21.3 ± 0.5 | 21.6 ± 0.4 | 20.2 ± 0.0 | 18.8 ± 0.5 | 21.1 ± 0.8 | 10.5 ± 0.6 | 9.7 ± 0.6 |
| SO-SMI-9702 | Ancient | SMI | 18.9 ± 0.0 | 9.7 ± 0.1 | 11.6 ± 0.5 | 19.2 ± 0.1 | 18.7 ± 0.6 | 17.7 ± 0.3 | 17.7 ± 0.1 | 16.7 ± 0.1 | 19.6 ± 0.3 | 10.8 ± 0.3 | 9.4 ± 0.3 |
| SO-SMI-10636 | Ancient | SMI | 20.6 ± 0.4 | 9.8 ± 0.4 | 10.4 ± 0.2 | 18.8 ± 0.1 | 18.3 ± 0.2 | 18.0 ± 0.2 | 18.7 ± 0.1 | 17.3 ± 0.0 | 19.7 ± 0.0 | 9.5 ± 0.1 | 8.7 ± 0.7 |
| SO-SMI-11080 | Ancient | SMI | 20.5 ± 0.3 | 10.3 ± 0.4 | 10.0 ± 0.6 | 20.1 ± 0.5 | 19.7 ± 0.2 | 19.0 ± 0.4 | 18.4 ± 0.0 | 18.6 ± 0.2 | 21.5 ± 0.4 | 10.0 ± 0.8 | 10.3 ± 0.2 |
| SO-SMI-257-12400 | Ancient | SMI | 21.0 ± 0.1 | 11.3 ± 0.2 | 10.5 ± 0.5 | 20.2 ± 0.1 | 19.5 ± 0.1 | 19.7 ± 0.0 | 18.7 ± 0.1 | 17.5 ± 0.0 | 20.1 ± 0.2 | 8.7 ± 0.2 | 8.7 ± 0.3 |
| SO-SNI-001 | Ancient | SNI | 21.9 ± 0.4 | 13.4 ± 0.1 | 12.6 ± 0.0 | 21.1 ± 0.2 | 20.4 ± 0.1 | 20.8 ± 0.5 | 20.6 ± 0.2 | 18.9 ± 0.2 | 21.2 ± 0.3 | 10.7 ± 0.5 | 10.4 ± 0.6 |
| SO-SNI-006 | Ancient | SNI | 20.6 ± 0.4 | 12.2 ± 0.2 | 13.1 ± 1.0 | 20.9 ± 0.6 | 19.7 ± 0.2 | 20.3 ± 1.0 | 20.8 ± 0.2 | 19.4 ± 0.4 | 21.1 ± 0.3 | 11.9 ± 0.4 | 8.8 ± 0.7 |
| SO-SNI-008 | Ancient | SNI | 20.4 ± 0.2 | 11.7 ± 0.3 | 13.1 ± 0.1 | 19.3 ± 0.2 | 19.3 ± 0.6 | 18.7 ± 0.8 | 19.3 ± 0.0 | 18.8 ± 0.2 | 20.8 ± 0.6 | 12.8 ± 0.2 | 11.9 ± 0.5 |
| SO-SNI-012 | Ancient | SNI | 20.1 ± 0.5 | 11.9 ± 0.6 | 13.3 ± 0.9 | 19.3 ± 0.9 | 19.5 ± 0.8 | 18.5 ± 0.1 | 19.4 ± 0.3 | 19.4 ± 0.2 | 21.0 ± 0.5 | 12.6 ± 0.6 | 11.4 ± 0.5 |
| SO-MBY-7064-14 | Modern | MBY | 19.6 ± 0.6 | 11.2 ± 0.1 | 11.4 ± 0.6 | 19.4 ± 0.7 | 18.0 ± 0.4 | NA | 18.1 ± 0.2 | 18.5 ± 0.1 | 21.2 ± 0.3 | 10.6 ± 0.5 | 8.6 ± 0.6 |
| SO-MBY-5204-08 | Modern | MBY | 19.9± 0.3 | 11.0 ± 0.4 | 10.0 ± 0.4 | 20.7 ± 0.2 | 18.7 ± 0.4 | 17.0 ± 0.3 | 18.0 ± 0.2 | 16.9 ± 0.5 | 20.1 ± 0.5 | 11.1 ± 0.0 | 7.8 ± 0.6 |
| SO-MBY-4923-07 | Modern | MBY | 22.0 ±0.4 | 11.2 ± 0.2 | 11.7 ± 0.3 | 22.5 ± 0.5 | 21.5 ± 0.5 | 21.0 ± 0.1 | 21.9 ± 0.3 | 21.2 ± 0.3 | 23.2 ± 0.2 | 10.1 ± 0.5 | 10.3 ± 0.6 |
| SO-MBY-5054-07 | Modern | MBY | 20.2 ± 0.3 | 11.8 ± 0.2 | 11.8 ± 0.1 | 21.4 ± 0.1 | 19.4 ± 0.2 | 19.2 ± 0.8 | 18.1 ± 0.1 | 19.4 ± 0.5 | 20.8 ± 0.1 | 10.4 ± 0.0 | 9.9 ± 0.1 |
| SO-MBY-5818-10 | Modern | MBY | 22.5 ± 0.3 | 10.5 ± 0.4 | 11.4 ± 0.2 | 23.1 ± 0.3 | 22.0 ± 0.4 | 22.6 ± 0.5 | 20.7 ± 0.1 | 20.8 ± 0.6 | 23.9 ± 0.3 | 11.2 ± 0.1 | 9.1 ± 0.3 |

**APPENDIX 6.**

**Boxplots comparing δ^15^N (top panel) and δ^13^C (bottom panel) values among archaeological sites in each region.**

****Colors represent different regions: red = Año Nuevo, olive = Monterey Bay, green = San Louis Obispo, blue = San Miguel Island, pink = San Nicolas Island.
